# A Single Neonatal Injection of Ethinyl Estradiol Impairs Passive Avoidance Learning and Reduces Expression of Estrogen Receptor α in the Hippocampus and Cortex of Adult Female Rats

**DOI:** 10.1371/journal.pone.0146136

**Published:** 2016-01-07

**Authors:** Tatsuomi Shiga, Takahiro J. Nakamura, Chiaki Komine, Yoshikuni Goto, Yasushi Mizoguchi, Midori Yoshida, Yasuhiko Kondo, Maiko Kawaguchi

**Affiliations:** 1 School of Agriculture, Meiji University, Kawasaki, Kanagawa, Japan; 2 Faculty of Pharmaceutical Sciences, Teikyo Heisei University, Tokyo, Japan; 3 Division of Pathology, National Institute of Health Sciences, Tokyo, Japan; 4 School of Life and Environmental Sciences, Teikyo University of Science, Tokyo, Japan; University of Barcelona, Faculty of Biology, SPAIN

## Abstract

Although perinatal exposure of female rats to estrogenic compounds produces irreversible changes in brain function, it is still unclear how the amount and timing of exposure to those substances affect learning function, or if exposure alters estrogen receptor α (ERα) expression in the hippocampus and cortex. In adult female rats, we investigated the effects of neonatal exposure to a model estrogenic compound, ethinyl estradiol (EE), on passive avoidance learning and ERα expression. Female Wistar-Imamichi rats were subcutaneously injected with oil, 0.02 mg/kg EE, 2 mg/kg EE, or 20 mg/kg 17β-estradiol within 24 h after birth. All females were tested for passive avoidance learning at the age of 6 weeks. Neonatal 0.02 mg/kg EE administration significantly disrupted passive avoidance compared with oil treatment in gonadally intact females. In a second experiment, another set of experimental females, treated as described above, was ovariectomized under pentobarbital anesthesia at 10 weeks of age. At 15–17 weeks of age, half of each group received a subcutaneous injection of 5 μg estradiol benzoate a day before the passive avoidance learning test. Passive avoidance learning behavior was impaired by the 0.02 mg/kg EE dose, but notably only in the estradiol benzoate-injected group. At 17–19 weeks of age, hippocampal and cortical samples were collected from rats with or without the 5 μg estradiol benzoate injection, and western blots used to determine ERα expression. A significant decrease in ERα expression was observed in the hippocampus of the estradiol-injected, neonatal EE-treated females. The results demonstrated that exposure to EE immediately after birth decreased learning ability in adult female rats, and that this may be at least partly mediated by the decreased expression of ERα in the hippocampus.

## Introduction

Xenoestrogens are compounds in the environment that mimic the physiologic activity of estrogen; they are contained in industrial contaminants, plastics or plasticizers, pesticides, and certain plants [1,2]. By binding to estrogen receptors (ERs), these compounds can disturb homeostatic responses in the endocrine system [1,3]. In fact, exposure to such estrogenic substances can have a profound adverse influence on the development of the nervous system in both animals and humans. One such influence is the impairment of learning and memory [4–9]. For example, the female offspring of Wistar rat dams exposed during pregnancy and lactation to bisphenol A (BPA), an estrogenic agent in polycarbonate plastics, demonstrated impaired learning in step-down passive avoidance tasks as adults [6]. Additionally, the female progeny of dams exposed from gestation to lactation to the estrogenic agent isobutylparaben, a widely used preservative, demonstrated poor social recognition performance [7]. Notably, the heavy metal pollutant cadmium can also mimic estrogens [10], such that cadmium toxicity can inhibit avoidance acquisition in female offspring [9].

The mechanism through which these toxic effects are induced may involve changes in hippocampal ERα expression after maturation. In female rodents, acute estrogen treatment enhanced hippocampus-dependent learning behaviors such as avoidance and spatial memory [11–13]. At the molecular level, however, little is known about the effect of perinatal xenoestrogen exposure on hippocampal or cortical ERα expression, although many reports have demonstrated altered ERα expression in the hypothalamus [14–16]. Kundakovica et al. demonstrated that exposure to 20 μg/kg BPA during lactation reduced ERα expression in the prefrontal cortex, but not in the hippocampus, in intact female mice [17]. BPA, however, also disturbs thyroid activity, so it remains unclear whether the reduced ERα expression was specifically induced by the estrogenic activity of BPA. In addition, neonatal exposure to estrogenic compounds can affect gonadal development and subsequent blood estrogen levels after maturation [18], and most studies cannot exclude this indirect effect on the brain and behavior. Therefore, there is a gap in our understanding of how the amount and timing of xenoestrogen exposure directly affects learning behavior and/or ERα expression in the hippocampus and cortex.

In this study, our objective was to determine whether a single neonatal dose of a xenoestrogen, and if so what dose, would directly affect learning behavior and ERα expression in the hippocampus and cortex. We selected ethinyl estradiol (EE), a constituent of contraceptives, as a model compound. Because EE does not bind to α–fetoprotein, it is transported to the brain and excreted from the body within 24 h after a subcutaneous injection, thus limiting its exposure period [19,20]. Rats were exposed to a low dose (0.02 mg/kg EE; LEE), that was chosen based on a study reporting early onset of persistent estrus from 14 weeks of age in rats that received a single neonatal injection of EE [20]; or a high dose (2 mg/kg EE; HEE) that was selected based on data we collected previously, in which sexual behavior in rats was inhibited by a single injection of EE (unpublished data, Maiko Kawaguchi). In addition, 20 mg/kg 17β-estradiol (E2) was chosen as a comparison based on a previous study reporting the loss of sexual differentiation of the sexually dimorphic nucleus of the preoptic area (POA) following E2 exposure [21]. For behavioral testing, we selected the passive avoidance test, which has been validated for estrogen sensitivity [11]. The passive avoidance test is also known to be affected by perinatal estrogenic agents in females [6], and has been shown to utilize anatomical substrates including the hippocampus [22,23], and its associated cortex [24]. In addition, the direct effects of EE were tested by ovariectomizing (OVX) one group of females and providing a controlled replacement does of estrogen to half the group.

## Materials and Methods

### Animals

Pregnant Wistar-Imamichi rats were obtained from the Institute for Animal Breeding Research (Ibaraki, Japan). Animals were maintained under controlled air conditions (room temperature [RT] 23 ± 1°C; humidity 50% ± 15%) with food and water available ad libitum, under a 12/12 h light/dark cycle with a light intensity of 200–300 lux. All procedures were approved by the Animal Care and Use Committee of Meiji University of Agriculture (approval ID#: IACUC11-0015).

### Treatments and test schedule

Eight offspring per litter were selected within 24 h after birth. Subsequently, female pups were subcutaneously injected with one of the following: 0.02 mg/kg EE (Tokyo Chemical Industry, Tokyo, Japan; LEE), 2 mg/kg EE (HEE), 20 mg/kg E2 (Sigma-Aldrich, St. Louis, MO, USA), or vehicle only (sesame oil, Sigma-Aldrich). They were weaned at the age of 3 weeks. In the first experiment, at 6 weeks of age, the rats underwent the passive avoidance test while gonadally intact, regardless of their estrus stage. Because neonatal exposure to xenoestrogens is known to affect both the gonads and brain, we next generated groups designed to investigate the specific effects on the brain. For this, another set of animals was divided into the same neonatal injection groups described above, then surgically ovariectomized (OVX) and treated with or without estradiol benzoate (5 μg/0.1 ml; EB). The OVX with EB group modeled normal fertile females, while the OVX without EB group modeled menopause. Females of all groups were OVX under pentobarbital anesthesia (40 mg/kg) at 10 weeks of age. At 15–17 weeks of age, 24 h before the passive avoidance test, half of the OVX females from all injection groups were injected with 5 μg/0.1 ml EB (Sigma-Aldrich). Two weeks later, 24 h prior to brain sampling for western blotting, the same females were again injected with 5 μg/0.1 ml EB. Passive avoidance testing was performed in quadruplicate in both the OVX with and without EB groups (n = 6–10/group). Six OVX animals per group were randomly chosen after the first set of behavioral tests for subsequent brain tissue collection for western blotting.

### Passive avoidance test

All rats were trained for the passive avoidance test. The step-through type passive avoidance test unit (PA-2010A & PAA-3001; O’ Hara & Co., Tokyo, Japan) comprised two compartments: bright and dark (500 × 150 × 270 mm). An automated guillotine door was used to isolate the compartments. The passive avoidance test consisted of acquisition and testing phases. During acquisition, rats were placed in the bright compartment with the door opened. After the rat moved into the dark compartment (all rats voluntarily moved within 100 s), the door was closed to restrict the rats to the dark compartment. Then, they were given a mild electric shock (0.3 mA for 3 s) through the floor grid. Infrared sensors monitored movement from the bright compartment to the dark compartment, which was recorded as transfer latency time in seconds. The testing phase was carried out 24 h after the acquisition phase. Transfer latency was recorded, and if the rats did not enter the dark compartment within 100 s, this was recorded as “no response.” The apparatus was cleaned with 70% ethanol solution before every test.

### Brain sample collection

**Fig 1:**
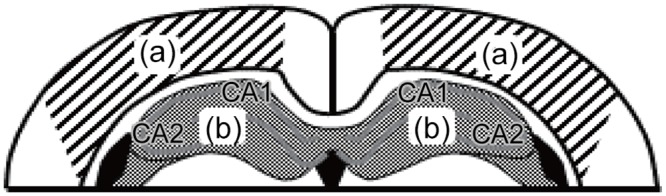
Illustration of a coronal section of the brain showing tissue sampling sites for the cortex (a) and hippocampus (b) [25]. CA1: CA1 region of the hippocampus; CA2: CA2 region of the hippocampus.

In the second experiment, we also examined the expression levels of ERα in the cortex and hippocampus of EE-treated OVX rats with or without EB. At 17–19 weeks of age, rats were sacrificed by deep ether anesthesia, and their brains removed. The brains were placed in chilled saline and sliced coronally at a thickness of 1 mm using a metal brain slicer (Muromachi, Tokyo, Japan). The cortex (Fig 1a) and hippocampus (Fig 1b) were isolated from the slice using scalpels under a stereomicroscope and stored at -80°C for processing. For homogenization, tissues were dispensed in 1 μl/mg lysis buffer containing mammalian protein extraction buffer (GE Healthcare, Connecticut, USA) and a 1% inhibitor cocktail (Thermo Scientific, Massachusetts, USA). Tissues were homogenized and sonicated on ice for 2 × 5 s each. Samples were centrifuged for 10 min at 14000 rpm at 4°C, and their supernatants were collected. The protein concentrations were determined using a 2-D Quant kit (GE Healthcare). The samples were mixed with a one-sixth volume of 62.5 mM Tris-HCl, pH 6.8, 10% glycerol, 5% 2-mercaptoethanol, 2.5% SDS, and 0.01% bromophenol blue, boiled for 5 min, and stored at -80°C.

### Western blotting

Frozen samples (40 μg protein) were separated on a 5–15% SDS-polyacrylamide gradient gel (BIO CRAFT, Tokyo, Japan) at 40 mA for 3 h. Molecular weight markers (Dual Color; BIO RAD, California, USA) were included in the run. Proteins were then transferred to polyvinylidene difluoride (PVDF) membranes overnight at 30 V. The membranes were blocked with 5% skim milk in TBS-T (100 mM Tris, 2.0% NaCl pH 7.5, 1% Tween-20) for 60 min at RT. The membranes were incubated overnight at 4°C with the ERα rabbit polyclonal antibody (MC-20, 1:500 dilution; Santa Cruz Biotechnology, Santa Cruz, CA) diluted in TBS-T. Protein loading was normalized to GAPDH using a monoclonal primary antibody (6C5, 1:5000 dilution; Santa Cruz Biotechnology). The membranes were washed three times with TBS-T for 5 min each and then incubated with the anti-rabbit (W401B, 1:10000 dilution; Promega, Wisconsin, USA) or anti-mouse secondary antibody (W402, 1:10000 dilution; Promega) for 1 h at RT. Antibody staining was detected using the enhanced chemiluminescence kit (ECL prime; GE Healthcare). The signals in developed images were quantified using ImageJ software (NIH, USA). The results are expressed as intensity of the signals in arbitrary densitometry units after normalization to GAPDH as an internal standard. Western blot analyses were done separately for the EB (-) and EB (+) injected groups due to the equipment’s limited sample capacity.

### Statistical analysis

Results of the passive avoidance test were analyzed by Kaplan-Meier survival analysis, followed by log-rank comparison. For other measures, one-way analyses of variance (ANOVA) and *post-hoc* Tukey-Kramer tests were used to compare multiple groups. Results of the ANOVAs are presented as the mean ± SEM. All results were considered significant at *P* < 0.05.

## Results

### Avoidance learning in EE-treated rats

**Fig 2:**
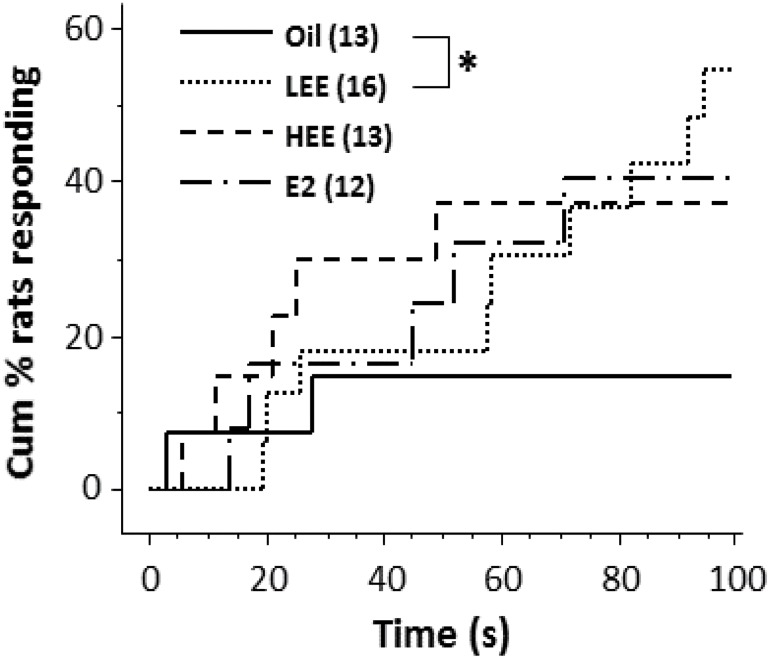
Impairment of avoidance learning in gonadally intact low dose ethinyl estradiol (LEE)-treated female rats. The line plot shows effects of LEE (0.02 mg/kg), HEE (2 mg/kg), and 17β-estradiol (E2; 20 mg/kg) treatment within 24 h after birth on the cumulative percentage of rats that displayed a transfer response in the passive avoidance test. Six-week-old gonadally intact animals were used in this experiment. Numerals in parentheses indicate the number of rats in each group. * *P* < 0.05, log-rank comparison.

**Fig 3:**
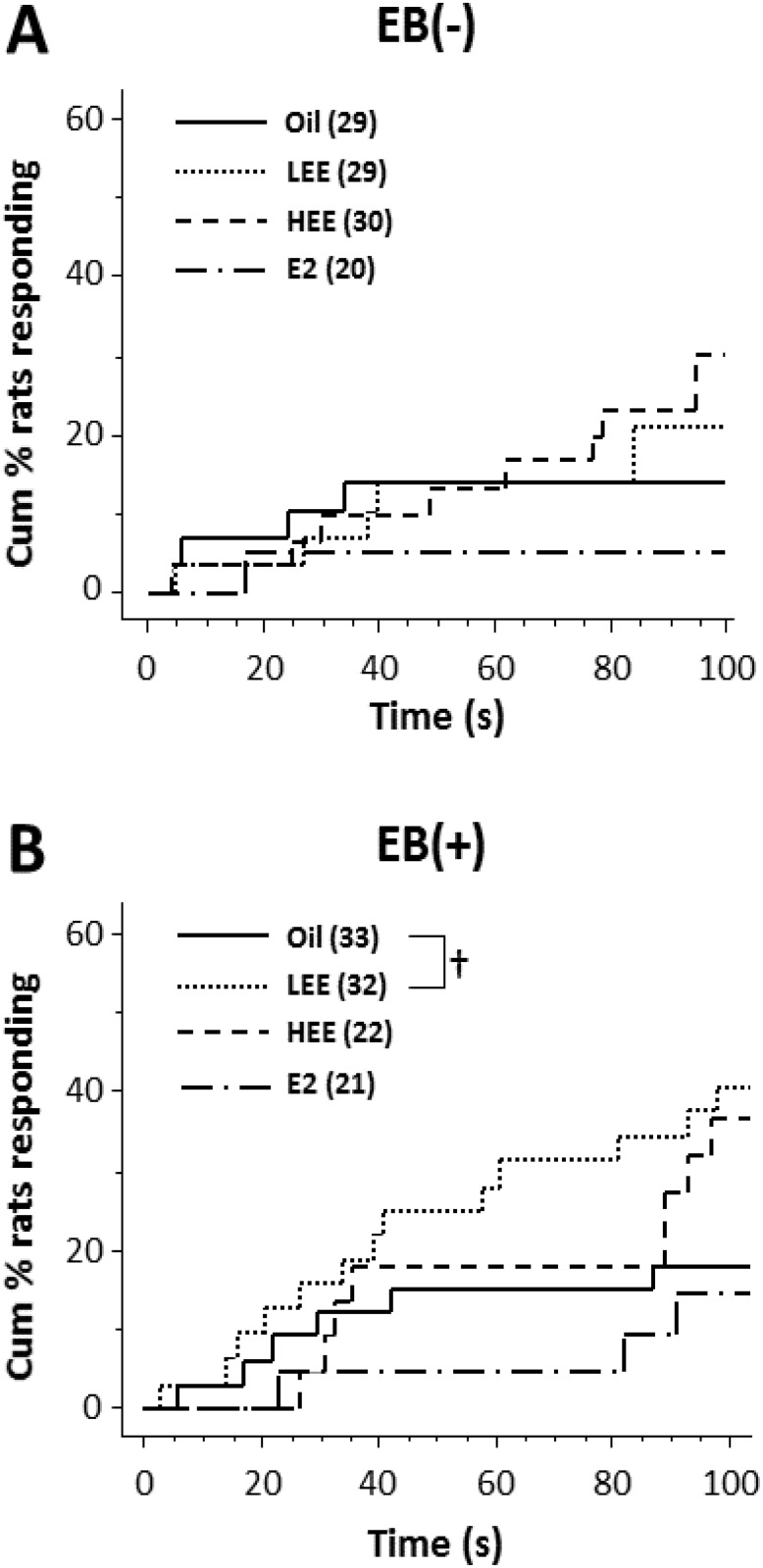
Impairment of avoidance learning in ovariectomized (OVX) low dose ethinyl estradiol (LEE)-treated female rats. The line plots show the effect of LEE (0.02 mg/kg), HEE (2 mg/kg), and 17β-estradiol (E2; 20 mg/kg) treatment within 24 h after birth on the cumulative percentage of rats that displayed a transfer response in the passive avoidance test. All rats were OVX at 10 weeks of age; at 15–17 weeks, the animals were either not injected (A) or injected with EB (B) 1 day before the session. Numerals in parentheses indicate the number of rats in each group. †*P* < 0.1, log-rank comparison.

The results of the passive avoidance test are shown as survival curves in Figs 2 and 3, demonstrating the time-course change in the cumulative percentage of animals producing a transfer response (also see S1A–S1C Fig). The LEE-treated rats showed a shorter latency to enter the dark/shock compartment in the first (gonadally intact) experiment (Oil vs. LEE; *P* < 0.05, log-rank comparison; Fig 2). In the second experiment, no significant differences were found between groups in the non-EB-injected condition (Fig 3A). On the other hand, in the EB-injected condition, the LEE-treated rats again showed a tendency toward a shorter latency to enter the shock compartment (Oil vs. LEE; *P* = 0.057, log-rank comparison; Fig 3B).

### Effects of neonatal exposure to EE on expression levels of ERα in the cortex and hippocampus

**Fig 4:**
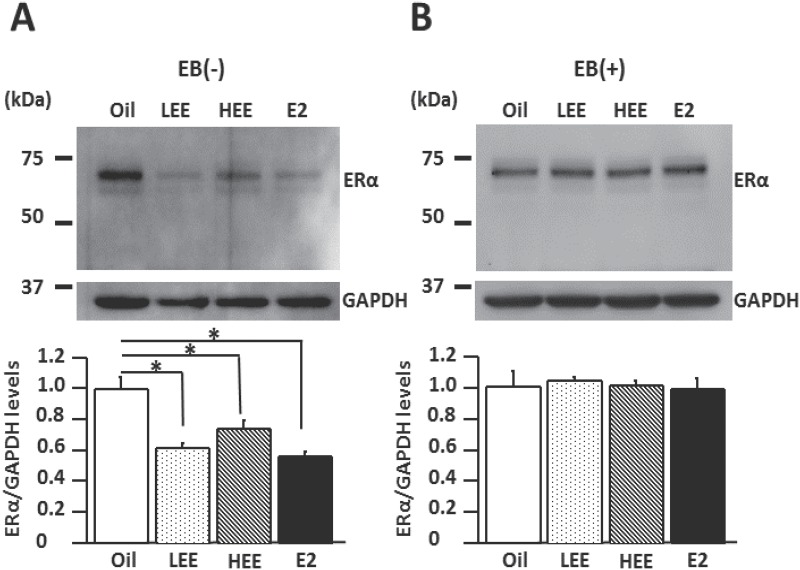
Neonatal exposure to ethinyl estradiol (EE) decreased the expression level of estrogen receptor alpha (ERα) in the female rat cortex. A. Representative western blot images showing the ERα expression in the cortex of EE-treated 15–18-week-old ovariectomized (OVX) rats injected with estradiol benzoate (+) or without injection (-). B. The levels of ERα in the cortex. *P < 0.05, Tukey-Kramer test; n = 6/group.

ERα protein levels in the cortex of LEE-, HEE-, and E2-treated females were significantly lower than in oil-treated females in the non-EB-injected group (Fig 4A; *P* < 0.001, *P* < 0.01, and *P* < 0.001, respectively). In contrast, with EB estrogen replacement, there was no significant difference in ERα protein levels in the cortex among the neonatal treatment groups (Fig 4B).

**Fig 5:**
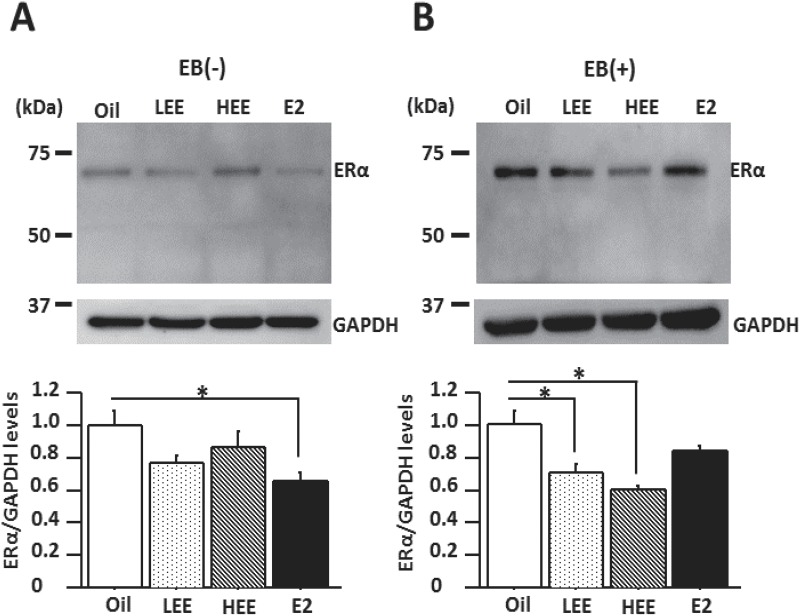
Neonatal exposure to ethinyl estradiol (EE) decreases the expression level of estrogen receptor alpha (ERα) in the hippocampus of adult female rats. A. Representative western blot images showing ERα expression in the hippocampus of EE-treated ovariectomized OVX rats injected with (+) estradiol benzoate or without injection (-) EB. B. Levels of ERα expression in the hippocampus. *P < 0.05, Tukey-Kramer test; n = 6/group.

ERα protein levels in the hippocampus of E2-treated females were significantly lower than in oil-treated females (Fig 5A; *P* < 0.05) in the non EB-injected group. In the EB-injected group, ERα protein levels in the hippocampus of LEE- and HEE-treated females were significantly lower than in oil-treated females (Fig 5B; *P* < 0.01 and *P* < 0.001, respectively).

## Discussion

The results of this study show that a single exposure to 0.02 mg/kg of EE within 24 h after birth lowered performance in the passive avoidance learning test. Additionally, a single exposure to 0.02 mg/kg and 2 mg/kg of EE within 24 h after birth reduced the expression levels of ERα in the hippocampus in the OVX/EB group, and in the cortex in the OVX/No EB group.

A single injection of LEE within 24 h after birth, which terminates the estrus cycle early after sexual maturation [20], induced a shorter latency to enter the dark/shock compartment in the passive avoidance-learning test in gonadally intact female rats. The results of this study are similar to those that demonstrated impaired learning performance in the passive avoidance test in female mice pre- and postnatally exposed to 40 μg/kg/day BPA [6]. Unlike that study, though, we limited off-target (i.e., non-estrogenic) disruptive effects by using the rapidly metabolized and excreted estrogenic agent EE. Thus, our data strongly suggest that exposure to a single dose of EE within 24 h after birth reduced avoidance learning through an estrogen-mediated process. This study also reveals a tendency towards shorter latencies to enter the shock compartment in the passive avoidance learning test of the LEE OVX group that received EB after maturation (on the day before the passive avoidance learning test) as a normal/fertile female model. In using OVX groups, we showed that some of the learning impairment in the intact female was caused by a direct action of neonatal EE on the brain, and that this effect was stronger in the presence of circulating estrogen. Further, this study also indicates that a high dose of an estrogenic agent is not necessarily more potent than a low dose. The learning behavior was affected by a dose lower than the level at which sexual behavior was affected in our previous experiments (see the Introduction). In the general dose-response model, an effect increases with increasing dose; however, estrogen-like substances have been reported to show an inverted U-shape curve, wherein the effect is greater at low-moderate doses than at high doses [26,17,27]. This study also indicates that the effect of exposure to low-dose estrogen-like substances during critical developmental periods can persist late into life.

In the hippocampus, expression levels of ERα were significantly reduced in the EE-exposure group only when EB was injected 24 h before brain sampling; therefore, the injection of estradiol may have acted to trigger a latent effect of neonatal EE exposure on the hippocampus. This study demonstrates for the first time that a single exposure within 24 h after birth affects ERα in the hippocampus, even after maturation, in the normal fertile female model, but not the menopause model. These results also highlight the importance of ovarian estrogen in both reproductive and normal daily brain function. Estrogen increases dendritic spine density on pyramidal cells, as well as N-methyl-D-aspartate (NMDA) receptor expression. It also enhances the magnitude of long-term potentiation (LTP) through ERα [11–13]. In females, it also improves performance on hippocampus-dependent learning tasks such as passive avoidance [11,13]. Along with the trend towards a shorter latency to enter the shock compartment of the OVX and EB-injected LEE group in the passive avoidance learning test, the lower performance in the passive avoidance learning test in the present study could have been partly caused by the reduction of ERα expression in the hippocampus. On the other hand, the passive avoidance test was only affected in the LEE group, though ERα expression in hippocampus was affected in both the LEE and HEE groups, and in the fertile female model. This difference between the results of learning behavior and ERα expression suggest possible compensatory changes that occurred in other regulatory systems, such as ERβ expression, synaptic modification, or hippocampal NMDA receptor expression [28,12,29]. In future studies, it will be necessary to consider such variables. Furthermore, in the menopause model, a single injection of EE within 24 h after birth had no effect on the expression levels of ERα in the hippocampus after maturation. The potential confound of ovarian estrogen production, and possibly position in the estrus cycle, will also need to be addressed in future work.

In contrast to the hippocampus, EE exposure reduced ERα expression in the cortex in the menopause model. On the other hand, no significant difference was observed in cortical ERα expression between the groups that received EB injections 24 h before brain sampling. Therefore, EB injection 24 h before brain sampling apparently masked the effect of the single neonatal exposure to EE on the reduction of ERα expression in the cortex. Exposure to estrogenic agents within 24 h after birth reduced ERα expression after maturation, and changed the response to estrogen; its effect may have varied in a site-specific manner. In the study conducted by Andrea Gore and colleagues in 2011 [14], sequential exposure to EB (1 mg/kg) and methoxychlor (100 mg/kg) 7–19 days after birth also increased expression levels of ERα in the POA of female rats, while no change was found in the mediobasal hypothalamus. Here, we found that the effect of estrogen exposure during the developmental period varied between the hippocampus and cortex. These results re-affirm the importance and complexity of assessing the risk of exposure to estrogen-like substances during the developmental period.

## Supporting Information

S1 Fig Distribution of latencies for transfer response in the passive avoidance test. Each dot indicates the latency of the individual. A: gonadally intact, B: ovariectomized (OVX), C: OVX and replaced with estradiol benzoate (EB).
(PDF)
