# Supplementary figures and images for: A Single Neonatal Injection of Ethinyl Estradiol Impairs Passive Avoidance Learning and Reduces Expression of Estrogen Receptor α in the Hippocampus and Cortex of Adult Female Rats
Source: PLoS One. 2016 Jan 7;11(1):e0146136. doi: 10.1371/journal.pone.0146136 (PMC4712149; doi:10.1371/journal.pone.0146136)

S1 File: Supplemental Figures

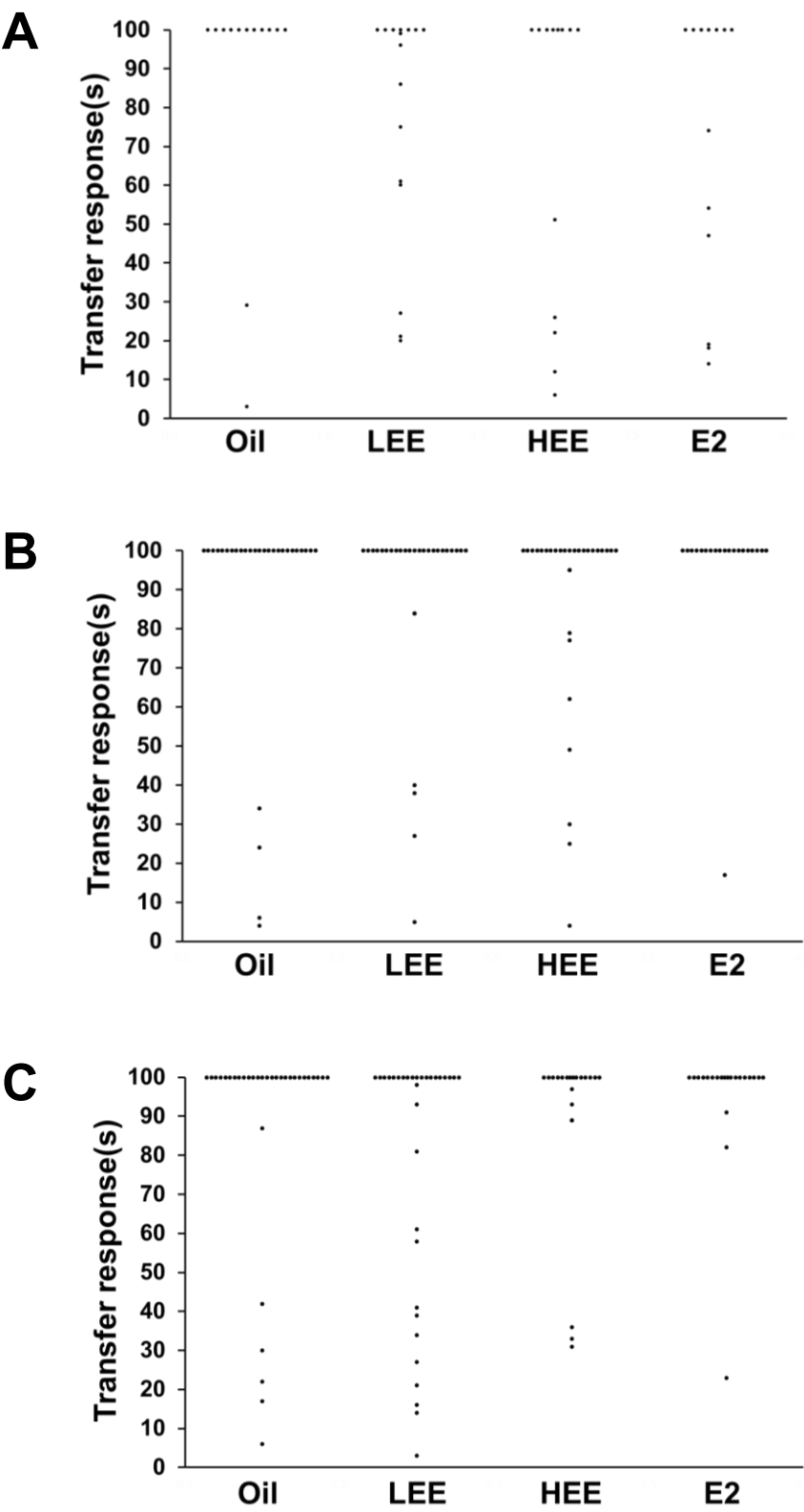

Supplement: S1 Fig — Each dot indicates the latency of the individual. A: gonadally intact, B: ovariectomized (OVX), C: OVX and replaced with estradiol benzoate (EB). (PDF) [file pone.0146136.s001.pdf]
